# Focus meets motivation: When regulatory focus aligns with approach/avoidance motivation in creative processes

**DOI:** 10.3389/fpsyg.2022.807875

**Published:** 2022-08-30

**Authors:** Christina Mühlberger, Paul Endrejat, Julius Möller, Daniel Herrmann, Simone Kauffeld, Eva Jonas

**Affiliations:** ^1^Department of Psychology, Social Psychology, University of Salzburg, Salzburg, Austria; ^2^Faculty of Psychology and Human Movement Science, Industrial and Organizational Psychology, University of Hamburg, Hamburg, Germany; ^3^Department for Pedagogy and General Didactics, Institute for Educational Sciences, Technical University of Braunschweig, Braunschweig, Germany; ^4^Institute of Psychology, Industrial/Organizational and Social Psychology, Technical University of Braunschweig, Braunschweig, Germany

**Keywords:** self-regulation, regulatory focus/fit, approach/avoidance motivation, creative performance, creative experience, system vs. strategy

## Abstract

According to Regulatory Focus Theory, two systems determine our strategies to pursue goals – the promotion and the prevention system. Individuals with a dominant promotion system focus on achieving gains, i.e., promoters, and individuals with a dominant prevention system focus on avoiding losses, i.e., preventers. Regulatory Fit Theory suggests that a fit between this focus and the situation causes superior performance and makes individuals feel right. We transfer the fit idea to the interaction of dominant regulatory focus (promotion vs. prevention) with motivational direction (approach vs. avoidance motivation). We investigated these interaction effects on individuals’ performance and their experience within creativity workshops. In Study 1 (*N*_1_ = 172), using multi-level analyses, we found that a promotion focus was associated with fluency and a prevention focus with elaborated ideas. This effect was stronger, when preventers also scored high on avoidance motivation. Further, preventers experienced more autonomy support and were more satisfied when they scored high on avoidance. Promoters high on approach motivation reported more autonomy support and more satisfaction than preventers high on approach motivation. For Study 2 (*N*_2_ = 112), we used an experimental design: After measuring regulatory focus, we manipulated approach vs. avoidance motivation in creativity workshops. Using multi-level analyses, we did not find main or interaction effects on fluency or elaboration but we found interaction effects on participants’ experience of the creativity workshop. Preventers were more satisfied when they received the avoidance condition. Promoters reported less autonomy support, lower satisfaction, and more perceived conflicts within their teams in the avoidance condition.

## Introduction

An insurance company wants to come up with new concepts to attract university students and convert them into loyal customers. For this purpose, it puts together a team of four employees: Alex, Beth, Caroline, and Kim. Alex and Beth were selected because they describe themselves as the ones who always try to achieve the “next-level” ideas, Caroline and Kim because they perceive themselves as the vigilant controllers who avoid that obvious mistakes are overseen and the project becomes shipwrecked. In order to come up with superior ideas, the company offers two different creativity workshops in which the employees get to know creativity methods. Alex and Kim participate in a workshop presenting methods that focus on the quantity of ideas. Caroline and Beth participate in a workshop presenting methods that focus on the detailedness of ideas. At the end of the project, only Alex and Caroline behave as expected: Alex is energized and determined to come up with good concepts, focuses on approaching superior ideas, and comes up with a lot of ideas. Caroline is worried about making mistakes, focuses on avoiding risks, addresses the thorny issues, and thinks each idea through. However, Beth and Kim do not perform well and complain about their work experience. With this article, we provide explanations for this finding.

Two factors that have been shown to influence task performance and experience are an individual’s regulatory focus and their motivational direction. While regulatory focus refers to ideal (*promotion focus*) or ought (*prevention focus*) selves (Higgins, 1997), *approach and avoidance motivation* capture basic psychological processes referring to the motivational urge to move toward or away from something (Elliot and Thrash, 2010; Harmon-Jones E. et al., 2013).

The two factors also play a crucial role in creative tasks. A promotion focus has been associated with generating many and original ideas (e.g., Crowe and Higgins, 1997; Herman and Reiter-Palmon, 2011) and a prevention focus with accurately evaluating the quality of ideas (Herman and Reiter-Palmon, 2011). There is also evidence that approach motivation increases creativity and avoidance motivation reduces creativity but increases attention to details (e.g., Friedman and Förster, 2002, 2005; Mehta and Zhu, 2009).

Next to these direct effects, we suggest that the interaction of regulatory focus with motivational direction should be considered when people work on a creative task. We thereby build on the regulatory fit idea claiming that a match between people’s regulatory focus and the means they choose to pursue a certain goal affects people’s experience of the current situation and their performance (Higgins, 2000, 2005). In the current paper, we investigate the fit between people’s dominant regulatory focus and their motivational direction. More concretely, we investigate whether a promotion focus and approach motivation together or a prevention focus and avoidance motivation together intensify the effect on individuals’ creative performance and affect their experience of a creativity workshop. In Study 1, we carried out 3-day design thinking workshops and in Study 2, we carried out 1-day creativity workshops. We used participants’ self-report data on their experience of the workshops as well as the number of ideas and the rated elaboration of the ideas in a creative task. Combining regulatory focus with the motivational directions approach and avoidance, we follow Scholer and Higgins (2008)’ suggestion that “It will be interesting in future work to consider how these two approaches might together suggest new ways in which to study approach and avoidance motivations in self-regulation.” (p. 499).

### Regulatory focus

Regulatory focus theory (RFT, Higgins, 1997) considers two fundamental self-regulatory systems – the promotion and prevention system – to be the basis for human goal-pursuit. The two systems determine our perception of the world and our preference for information and strategies to pursue goals. The promotion system focuses on growth and how one would ideally like to be (ideal-self) and thereby applies eager strategies. The prevention system focuses on security and how someone should be (ought-self) and thereby applies vigilant strategies. Obviously, individuals and teams need both systems because each uses essential strategies for effective goal pursuit, but individuals tend to follow a specific system more than the other (Higgins, 1997; Shah et al., 1998). In our example from the beginning, Alex and Beth have a dominant promotion focus and Caroline and Kim have a dominant prevention focus. Alex and Beth can hardly wait to come up with new concepts for the insurance company, looking forward to present creative and innovative ideas. Caroline and Kim worry about not performing well and thus, think through each idea in detail.

A dominant regulatory focus represents an immanent perspective on the world, differentiating between goals (gains/security) and anti-goals (stagnation/loss), and determining the desired goal point. Individuals with a dominant promotion focus (i.e., promoters) are oriented toward growth because they are more sensitive to gains than to loss. Their desired-end states are ideals, wishes, and hopes they try to achieve. In our example, Alex who is high on promotion has the desire for success in generating concepts (striving for gain). Individuals with a dominant prevention focus (i.e., preventers) are oriented toward security because they are more sensitive to loss than to gains. Their desired-end states are obligations and responsibilities they try to fulfill. In our example, Caroline who is high on prevention, has the goal of avoiding failure in generating concepts (avoiding loss). Regulatory focus not only works at the dispositional level, i.e., the system-level, but also at the strategic level. At the strategic level, eager promotion vs. vigilant prevention strategies are used to reach the goal. Thereby, individuals high on promotion engage in approach-related behaviors such as using all opportunities (e.g., generating as many ideas as possible). Individuals high on prevention engage in avoidance-related behaviors such as trying to avoid errors (e.g., often checking the ideas) (Scholer and Higgins, 2008, 2012).

### Approach and avoidance motivation

While regulatory focus is a construct of socialization resulting in a focus on the ideal or ought self, approach and avoidance motivation is a result of rudimentary biological processes representing an impel to behave. Thus, the self as a guide for regulation plays a crucial role in regulatory focus. In approach and avoidance motivation, the motivational urge to move into a specific direction, i.e., toward or away from stimuli, is important (Elliot and Thrash, 2010; Harmon-Jones E. et al., 2013). Whether individuals are approach or avoidance motivated depends on their personality but also on the situation. Thus, approach and avoidance motivation can be triggered by a trait or state (Harmon-Jones E. et al., 2013). When people are approach motivated, they feel capable, energized, powerful, and determined and have an impulse to move toward something (Carver and White, 1994; Harmon-Jones C. et al., 2011; Harmon-Jones E. et al., 2013; Greenaway et al., 2015). When people are avoidance motivated, they are anxious, worried, and vigilant for negative stimuli and have an urge to move away from something (McNaughton and Corr, 2004; Corr et al., 2013; Harmon-Jones E. et al., 2013).

An established indicator of approach vs. avoidance motivation is cerebral asymmetry. Comparing EEG alpha power between frontal areas of the left and right hemisphere, serves as an indicator of frontal asymmetry. Relative left frontal activity has been shown to be associated with approach motivation and relative right frontal activity with avoidance motivation. For example, individuals with greater relative right frontal activity reported more negative affect as a response to negative emotion-inducing films and less positive affect to positive emotion-inducing films (for a review, see Harmon-Jones et al., 2010). In addition, previous research has shown that trait measures of approach motivation were associated with resting, baseline left frontal activity, suggesting that traits motivate one to approach or avoid something (for a review, see Harmon-Jones E. et al., 2013). Approach or avoidance motivation can also be induced using situational factors, such as body gestures (Harmon-Jones E. et al., 2013), emotional expressions (Coan et al., 2001) or cognitive manipulations. For example, Roskes et al. (2012) induced approach motivation telling participants to find as many words as possible in a puzzle and induced avoidance motivation telling participants to miss as few words as possible in the puzzle.

Apart from EEG, there is also a behavioral measure used in research on approach and avoidance to assess frontal activity (e.g., Wilkinson et al., 2010; Naylor et al., 2015), i.e., the line bisection task (Jewell and McCourt, 2000). People’s biased perception to the right or left visual field when trying to mark the midpoint of horizontal lines are taken to reflect neural activity in the contralateral hemisphere (Nash et al., 2010). Nash et al. (2010) showed that resting left frontal activity in EEG was related to baseline line bisection bias, reflecting the line bisection task as a dispositional measure of approach-related motivation. In a further study, they also showed links between state left frontal activity in EEG and line bisection bias, indicating that the line bisection task can be used to assess situational approach-related motivation.

### Regulatory focus and motivational direction in creative work

Creativity is a combination of originality and effectiveness. Originality means that something is unusual, novel, or unique and effectiveness means that something is useful or appropriate. While effectiveness varies depending on what is being evaluated, originality is the fundamental requirement of creativity (Runco and Jaeger, 2012). Research has found that the more ideas individuals generated, the more original the ideas were (Paulus et al., 2011; Dippo and Kudrowitz, 2013; Kudrowitz and Wallace, 2013). Thus, fluency is a core criterion of the creative process, more concretely, of the process of divergent thinking. Divergent thinking refers to generating creative ideas. It requires people to explore new ways of thinking in order to generate multiple solutions to a problem. Two important facets of divergent thinking are fluency and elaboration (Guilford, 1957). Fluency is the rapid generation of ideas, where quantity is important but not quality. Elaboration refers to the amount of details of an idea (Guilford, 1957). It has been shown that fluency and elaboration are uniquely associated with important creativity outcomes (e.g., Dumas et al., 2021). Next to divergent thinking, there is convergent thinking indicating that people structure, organize, and reduce the solutions to come up with a clear solution (Guilford, 1956).

Previous research found an association between individuals with a strong promotion focus and the creativity factors fluency and originality: Promoters list more characteristics of an item (Crowe and Higgins, 1997) and generate more hypotheses about objects that are difficult to recognize than preventers (Liberman et al., 2001). Moreover, they generate more creative uses for a brick than preventers (Friedman and Förster, 2001). Even in teams, a collective promotion focus predicted idea generation and idea promotion, while a collective prevention focus did not (Rietzschel, 2011). The enhanced fluency of promoters appears to be due to their inclination to ensure “hits” and avoid omission (Crowe and Higgins, 1997) and their preference for change over stability (Liberman et al., 1999). Regarding speed, for example in drawing tasks or proofreading a text, promoters are faster than preventers (Förster et al., 2003). This can be explained by the fact that promoters construe information globally which means that they concentrate on the big picture rather than on the details (Lee et al., 2010).

Although there are no studies showing a direct association between individuals with a strong prevention focus and the creativity factor elaboration, research has found that preventers construe information locally which means that they predominantly concentrate on details rather than on the big picture (Lee et al., 2010). They are very conscientious and accurate (Friedman and Förster, 2001; Förster et al., 2003) and rather stick to fewer ideas (Crowe and Higgins, 1997) because they want to ensure against making mistakes (e.g., bad ideas). When it comes to proofreading a text, they find more difficult errors than promoters (Förster et al., 2003). The preventers’ focus on the details may help them to know what they should avoid in order to maintain security (Förster and Higgins, 2005).

Many tasks in our daily lives can be categorized as promotion- or prevention-tasks because they either require an eager strategy (e.g., generating many ideas) or a vigilant strategy (e.g., elaborating detailed ideas). However, there are also tasks where both regulatory styles are beneficial. These are tasks that require divisible components that allow people to adopt their specific strategies (Bohns and Higgins, 2011). For example, in a creative task, individuals high on promotion generate the ideas and individuals high on prevention work accurately, draw their attention to details, and can discover problems. This may lead to better outcomes and more satisfaction with the outcomes. Herman and Reiter-Palmon (2011) investigated the influence of regulatory focus on different phases of the creative process, namely the idea generation and idea evaluation phase. The results were in line with their hypothesis that within the idea generation phase, a high promotion focus is more beneficial to the generation of original, creative ideas which replicates previous research showing that promotion focus is associated with creativity (Friedman and Förster, 2001; Lam and Chiu, 2002; Van-Dijk and Kluger, 2011). For the evaluation phase, both foci were beneficial– promotion focus was positively related to participants’ ability to recognize the originality of the ideas and prevention focus predicted participants ability to recognize quality of the ideas. In other words, while for idea generation, only promotion focus was beneficial, for idea evaluation, both foci were important.

Regarding approach and avoidance motivation, there is also evidence that they affect the creative process. For example, when individuals were asked to perform body actions associated with approach motivation – i.e., arm flexion which is a motor action directed toward oneself – they generated more creative uses for a brick than when they were asked to perform bodily actions associated with avoidance motivation – i.e., arm extension which is a motor action directed away from oneself (Friedman and Förster, 2002, 2005). Moreover, approach motivation has been shown to enhance performance on tasks requiring originality while avoidance motivation has been shown to enhance performance on tasks requiring attention to details (Mehta and Zhu, 2009).

### Fit between regulatory focus and motivational direction and its effects

Although studies have shown that at the system-level, promotion and approach are positively correlated and prevention and avoidance are positively correlated (Amodio et al., 2004; Elliot and Thrash, 2010), in a confirmatory factor analysis, a four-factor solution provided the best fit. This solution separated promotion, prevention, approach, and avoidance temperament (Elliot and Thrash, 2010). This shows that regulatory focus and motivational direction differ: While promotion and prevention focus are based in socialization emphasizing ideal and ought standards (Higgins, 1997), approach and avoidance motivation are based in biology representing an impulse to move toward or away (Elliot and Thrash, 2010; Harmon-Jones E. et al., 2013). At the system level, the approach and avoidance systems are present in both the promotion and prevention system as people try to approach desired end-states and avoid undesired end-states (Amodio et al., 2004; Scholer and Higgins, 2008, 2012; Sassenberg and Vliek, 2019). This means that individuals high on promotion try to approach growth (+1) and to avoid non-growth (0). Individuals high on prevention try to approach safety (0) and to avoid loss or danger (−1). Transferred to our initial insurance example, the two promoters Alex and Beth try to approach showing a good performance (+1) and try to avoid not showing a good performance, i.e., stay where they are with their performance (0). The two preventers, Caroline and Kim try to approach not showing a bad performance (0) and try to avoid showing a bad performance (−1).

**FIGURE 1:**
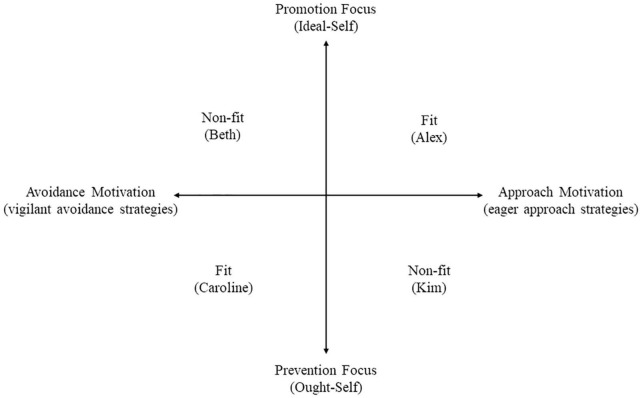
Interaction of dominant regulatory focus (promotion vs. prevention) with motivational direction (approach vs. avoidance motivation) and the fit or non-fit between the two variables.

Alex, Beth, Caroline, and Kim, get the same task (approaching the desired end-state of attracting university students and converting them into loyal customers) but only Alex and Caroline perform well. One reason for this is that individuals not only differ in regulatory focus but also in their motivational direction at a strategic-level. Here, individuals high on promotion and prevention select different strategies to approach their desired and avoid their undesired end-states. Promoters prefer to use eager approach strategies and preventers prefer to use vigilant avoidance strategies. In our initial insurance example, the two promoters Alex and Beth prefer approach strategies such as generating many ideas while the two preventers, Caroline and Kim prefer avoidance strategies such as avoiding to generate flawed ideas. The workshop presenting methods that focus on the quantity of ideas serves Alex’ promotion focus but not Kim’s prevention focus. The workshop presenting methods that focus on the detailedness of ideas serves Caroline’s prevention focus but not Beth’s promotion focus. Thus, Alex indeed generates many ideas and Caroline generates few but conscientiously elaborated ideas. The manner in which Alex and Caroline engage in this process, fits their underlying regulatory focus which makes them feel right. This is also known as regulatory fit (Figure 1).

Evidence for effects of regulatory fit has been found in a number of studies. In research on motivation and goal attainment, a study found that a promoter’s motivation and performance increases when the task incentive is framed as gaining or not gaining money. Reversed, a preventer’s motivation and performance increases when the task incentive is framed as losing or not losing money (Shah et al., 1998). Investigating a fit between people’s focus and their motivational state, Keller and Bless (2006) found that a promoter achieved better performance when the task induced approach motivation (approaching a good performance) and a preventer achieved better performance when the task induced avoidance motivation (avoiding a poor performance). Further, coaching sessions are more successful in terms of goal attainment, if promoters think about actions to approach a goal, whereas preventers benefit from thinking about which action should be avoided (Mühlberger et al., 2021). Interestingly, the positive effects of a fit condition are not limited to a specific situation but can also spill over to subsequent action. For instance, participants who were instructed to think about action plans which fit their focus, rated subsequent pictures more positive that those in the non-fit condition. Thus, the whole situation feels right and individuals transfer the value of the fit-experience to subsequent objects (Higgins et al., 2003).

A regulatory fit makes individuals “feel right” about what they are doing and they engage more strongly in this behavior (Higgins, 2005; Cesario and Higgins, 2008). It is experienced because using the preferred strategy sustains and does not disrupt the current regulatory focus (Higgins, 2005). A “feeling-right” is experienced as assigning an increased value to the current behavior (Higgins et al., 2003; Higgins, 2005; Cesario and Higgins, 2008). Individuals identify with these behaviors and thus, experience them as emerging from their self. This is also known as autonomous self-regulation (Sheldon and Elliot, 1999; Deci and Ryan, 2000). Autonomous self-regulation can be enhanced by taking the others’ perspectives und trying to understand their individual approaches (for an overview, see Deci and Ryan, 2000). This behavior is also known as autonomy support and has been shown to increase intrinsic motivation, learning, performance, and goal attainment (e.g., Black and Deci, 2000; Osbaldiston and Sheldon, 2003; Losch et al., 2016; Kanat-Maymon and Reizer, 2017). Getting back to our initial example, Alex and Caroline feel supported in their autonomy because the workshop leader equipped them with creativity strategies that fit their underlying regulatory focus. They may feel understood in their individual way of thinking. Beth and Kim do not feel supported in their autonomy because they received the wrong creativity strategies not fitting their regulatory focus.

In the current study, we build on regulatory fit and explore the interaction between people’s dominant regulatory focus and people’s motivational direction, on their performance in and experience of creative tasks.

### Hypotheses and overview of studies

The aforementioned research shows that individuals high on promotion apply eager strategies such as generating many ideas and individuals high on prevention apply vigilant strategies such as insuring against errors (Crowe and Higgins, 1997; Friedman and Förster, 2001; Lam and Chiu, 2002; Förster et al., 2003). Moreover, approach motivation has been associated with creativity and avoidance motivation with attention to details (Friedman and Förster, 2002, 2005; Mehta and Zhu, 2009). Replicating this research, we predict main effects of regulatory focus and motivational direction regarding creative performance. More concretely, individuals high on promotion focus and individuals high on approach motivation should show high fluency. Extending previous research, individuals high on prevention focus and individuals high on avoidance motivation should show high elaboration of ideas. We base this hypothesis on research showing that a prevention focus and avoidance motivation are related to conscientiousness and accuracy (Friedman and Förster, 2001, 2002, 2005; Förster et al., 2003; Förster and Higgins, 2005; Mehta and Zhu, 2009; Lee et al., 2010) and that a prevention focus is beneficial in a different phase of the creative process, i.e., when it comes to evaluating the quality of the ideas (Herman and Reiter-Palmon, 2011).

We predict the following main effects regarding individuals’ creative *performance*:

Hypothesis 1a: A promotion compared to prevention focus leads to an enhanced generation of ideas (*fluency*).

Hypothesis 1b: An approach compared to avoidance motivation leads to an enhanced generation of ideas (*fluency*).

Hypothesis 2a: A prevention compared to promotion focus leads to an enhanced elaboration of ideas (*elaboration*).

Hypothesis 2b: An avoidance compared to approach motivation leads to an enhanced elaboration of ideas (*elaboration*).

We investigate the interaction of regulatory focus and motivational direction following the idea of regulatory fit (Higgins, 2000, 2005). We predict that a simultaneous dominant promotion focus and approach motivation, or a simultaneous dominant prevention focus and avoidance motivation should represent a fit for individuals and thereby positively affect individuals’ creative *performance*:

Hypothesis 3: A simultaneous dominant promotion focus and approach motivation enhances individuals’ generation of ideas (*fluency*) compared to a simultaneous dominant promotion focus and avoidance motivation.

Hypothesis 4: A simultaneous dominant prevention focus and avoidance motivation enhances individuals’ *elaboration* of ideas (*elaboration*) compared to a simultaneous dominant prevention focus and approach motivation.

Research has shown that a fit leads people to feel right about a current situation which also triggers reactions to later, separate activities or objects. The whole situation feels right which leads people to evaluate the whole situation more positive (Higgins et al., 2003). Thus, we predict the following hypotheses regarding individuals’ *experience* of the creative situation:

Hypothesis 5: A simultaneous dominant promotion focus and approach motivation leads individuals to evaluate the whole creative process more favorably than a simultaneous dominant promotion focus and avoidance motivation. This shows up in more perceived *autonomy support* (Hypothesis 5a), more *satisfaction with the teamwork* (Hypothesis 5b), and lower experience of *conflicts* within the team (Hypothesis 5c).

Hypothesis 6: A simultaneous dominant prevention focus and avoidance motivation leads individuals to evaluate the whole creative process more favorably than a simultaneous dominant prevention focus and approach motivation. This shows up in more perceived *autonomy support* (Hypothesis 6a), more *satisfaction with the teamwork* (Hypothesis 6b), and lower experience of *conflicts* within the team (Hypothesis 6c).

We investigate our hypotheses in two studies. In Study 1, we measured regulatory focus as well as motivational direction. In Study 2, we measured regulatory focus and manipulated motivational direction. In both studies, we used the context of creativity workshops, such that participants were involved in a creative process while they were participating in the study.

### Transparency, openness, and statistical analyses

All data and analysis codes are available at https://osf.io/uhbcq/?view_only=4166764c01da44159cab6bd9f6ae8e51. Materials for the studies are available by emailing the corresponding author. Descriptive analyses, scale building, and correlations were performed in SPSS (version 27, IBM, Armonk, NY, United States) and the moderation analyses were conducted in R 4.02 using the package nlme (R Core Team, 2021; Pinheiro et al., 2022). Results are reported as significant when they reach a significance level of 5% and reported as “failed to reach the significance level of 5%” when they reach a significance level of 10%. Means, standard deviations, skewness, kurtosis, and the correlations between all variables for Study 1 and for Study 2 are shown in Tables 1, 2. Regarding the normal distribution, skewness and kurtosis values of our data are in an acceptable range (Byrne, 2010; Hair, 2010), expect of handedness in Studies 1 and 2 and relationship conflict in Study 2. Handedness shows a negative skewness in both studies which is not surprising as there are more right- than left-handed individuals. Relationship conflict shows a positive skewness and a positive kurtosis. This could be due to the fact that the teamwork in study 2 was short and therefore there were no or only few relationship conflicts within the teams.

**TABLE 1:** Means, standard deviations, skewness, kurtosis, and correlations between variables for Study 1.

Variable	*M*	*SD*	*Skewness*	*Kurtosis*	1	2	3	4	5	6	7	8	9	10	11
1. Chronic promotion focus	5.26	0.98	–0.95	1.32	–	–	–	–	–	–	–	–	–	–	–
2. Chronic prevention focus	4.04	1.12	0.01	–0.70	−0.36***	–	–	–	–	–	–	–	–	–	–
3. RFI	1.22	1.73	–0.17	–0.47	0.80***	−0.85***	–	–	–	–	–	–	–	–	–
4. MI	0.04	0.08	–0.32	–0.10	–0.1	0.08	–0.11	–	–	–	–	–	–	–	–
5. Fluency	8.16	3.11	0.49	–0.05	0.32***	–0.11	0.25**	–0.04	–	–	–	–	–	–	–
6. Elaboration	0.31	0.27	1.19	1.40	–0.05	0.09	–0.09	−0.21**	−0.28***	–	–	–	–	–	–
7. Perceived autonomy support	5.94	0.78	–1.10	1.65	0.14	−0.15*	0.18*	–0.03	0.10	0.03	–	–	–	–	–
8. Satisfaction	7.39	2.04	–1.19	1.38	0.09	–0.12	0.13	0.02	–0.10	0.05	0.35***	–	–	–	–
9. Relationship conflict	2.43	1.11	0.71	–0.38	–0.02	–0.09	0.05	–0.03	–0.01	–0.01	–0.07	−0.25**	–	–	–
10. Task conflict	3.64	0.98	–0.08	–0.73	–0.04	–0.02	–0.01	–0.02	–0.03	0.05	–0.11	−0.27**	0.67***	–	–
11. Handedness	54.88	37.66	–2.36	4.99	0.01	0.12	–0.07	0.01	0.10	–0.02	0.01	–0.07	0.03	–0.04	–

M and SD represent mean and standard deviation.

*p > 0.05, **p > 0.01, and ***p > 0.001.

**TABLE 2:** Means, standard deviations, skewness, kurtosis, and correlations between variables for Study 2.

Variable	*M*	*SD*	*Skewness*	*Kurtosis*	1	2	3	4	5	6	7	8	9	10	11
1. Chronic promotion focus	5.05	0.68	–0.47	0.21	–	–	–	–	–	–	–	–	–	–	–
2. Chronic prevention focus	5.07	0.76	–0.40	0.15	–0.002	–	–	–	–	–	–	–	–	–	–
3. RFI	–0.02	1.01	0.27	0.32	0.67***	−0.75***	–	–	–	–	–	–	–	–	–
4. MI	–0.34	0.95	0.18	–0.05	0.19	–0.15	0.23*	–	–	–	–	–	–	–	–
5. Fluency	7.22	3.53	1.02	2.55	0.23*	–0.09	0.22*	0.04	–	–	–	–	–	–	–
6. Elaboration	0.74	0.37	0.49	0.95	–0.12	0.00	–0.08	0.09	−0.43***	–	–	–	–	–	–
7. Perceived autonomy support	5.85	0.85	–0.61	–0.61	0.16	0.22*	–0.06	0.09	0.09	0.01	–	–	–	–	–
8. Satisfaction	8.14	1.79	–1.73	4.19	0.08	0.25**	–0.13	0.14	0.07	–0.02	0.23*	–	–	–	–
9. Relationship conflict	1.31	0.7	3.33	11.93	0.02	−0.20*	0.16	0.13	–0.06	0.06	–0.13	−0.37***	–	–	–
10. Task conflict	1.84	0.93	1.88	4.60	0.05	–0.15	0.15	0.04	–0.10	0.09	−0.24*	−0.42***	0.80***	–	–
11. Handedness	54.02	36.92	–2.25	5.08	–0.17	0.16	−0.23*	–0.03	–0.06	0.08	0.04	0.11	–0.004	0.02	–

M and SD represent mean and standard deviation.

*p > 0.05, **p > 0.01, and ***p > 0.001.

## Study 1

### Materials and methods

#### Procedure and sample

Data was collected from 181 participants who mainly studied STEM (science, technology, engineering, and mathematics) subjects at the Technical University of Braunschweig. The data was collected in 14 Design Thinking (DT) workshops, which included a total of 37 teams. Registration took place online as part of the interdisciplinary profiling, in which students can choose between different offers of action-related competence trainings. As data for dominant regulatory focus, dominant motivational direction, and team membership was missing for some participants, our final sample consisted of *N* = 172 working in 37 teams that were nested in 14 workshops. Participants were predominantly male (*N* = 123) and the age ranged from 19 to 38 years (*M* = 24.91; *SD* = 2.58).

Data was collected within the context of DT workshops. DT is a user-centered, team-based method to solve wicked-problems (Buchanan, 1992; Brown, 2008; Endrejat and Kauffeld, 2017). These problems constitute challenges that have no clear problem definition and lack a clear stopping rule, what means that a solution is not right or wrong, but rather matches predefined requirements better or worse (Rittel and Webber, 1973). The core element of DT is that recipients are actively involved. Instead of the trainer generating solutions, the recipients develop ideas and concepts and the trainer only accompanies this process. Such active involvement is important as it increases the probability that generated solutions are implemented. To provide students with the necessary competencies to cope with complex challenges, the 3-day DT workshops are integrated into the universities’ elective soft-skills curriculum for students from all subjects, who receive course credit for participation. Each workshop had on average 13 participants that were split up into two or three teams that worked independently on a given challenge. These challenges were introduced by project partners like the facility management (*“How might we enhance office space efficacy to reduce energy-usage?”*) to create a realistic learning environment where students can gain the competencies to solve real life issues.

The workshop concept is based on the *field guide to human centered design* (IDEO, 2015) that consists of the three process phases *inspiration*, *ideation*, and *implementation*. At the beginning of the first day, participants received an input about the DT mind-set and worked on several short exercises to become familiar with the DT working mode. Subsequently, during the inspiration phase, students learned methods to identify users’ key needs. These methods were applied for the remainder of the first day. The second day started with the ideation phase during which the teams generated solutions to meet users’ needs using various creative techniques. And the end of the second day and for the beginning of the third day, the teams moved to the implementation phase, which aimed to turn an idea into an innovation. The developed concepts and solutions were presented to the project partners at the end of the third day.

#### Measures

To create data collection as unobtrusive as possible, we handed out the questionnaires on different measurement points using paper and pencil questionnaires. At the beginning of the first workshop day we measured participants’ dominant regulatory focus, motivational direction, handedness, sex, and age. After the lunch break of day 2, we conducted the alternative use test (AUT) to examine subjects’ idea elaboration and fluency. At the end of the second day, we handed the questionnaires to measure conflict and the perceived learning climate (autonomy support), and asked participants to indicate their satisfaction with the outcome of the teamwork. At the end of day 3, we collected the idea evaluation of the experts^1^.

##### Dominant regulatory focus index

Promotion and prevention focus were measured using an abbreviated, eight-item (four items measuring promotion and prevention focus, respectively) scale developed by Sassenberg et al. (2012). Sample items include “My motto is ‘Nothing ventured, nothing gained”’ (promotion focus; α = 0.78) and “If I do not reach my goal, I am becoming nervous” (prevention focus; one item was excluded due to insufficient item-total correlations leaving three items for the prevention subscale with α = 0.69). Participants had to indicate on a 7-point scale ranging from 1 = *not at all* to 7 = *very much* in how far each statement applies to them. For the analyses, we built a regulatory focus index (RFI) for which we subtracted the prevention mean score from the promotion mean score (e.g., Keller and Bless, 2006; Cesario and Higgins, 2008; Hamstra et al., 2014). Positive values indicate a relatively higher dominant promotion than prevention focus. Although we are aware of the statistical and methodical implications of working with difference scores (Edwards, 1994), the benefits (e.g., comparability, interpretability, and pragmatism) of an RFI outweigh the costs.

##### Motivational index

Participants completed the line bisection task (Jewell and McCourt, 2000), a measure that can indicate cerebral asymmetry and thus approach or avoidance motivation (Nash et al., 2010). It has been used in previous studies to assess approach and avoidance motivational processes (e.g., Wilkinson et al., 2010; Naylor et al., 2015). The task involved 8 staggered horizontal lines of different lengths on a sheet of paper and participants were instructed to mark the middle of each line. We calculated the distance of participants’ marks from the objective midpoint with rightward errors scored as positive values and leftward errors scored as negative values. By averaging the scores across the 8 lines, we built the mean of the line bisection score (*M* = 0.04, *SD* = 0.08, α = 0.76) indicating a motivational index (MI) with positive scores indicating relatively more rightward errors and thus, approach motivation.

##### Fluency and elaboration

We used a task from the alternative use test (AUT; Guilford et al., 1960) to measure *elaboration* and *fluency*. We asked each participant to generate as many alternative uses for a paperclip as possible within 3 min. Fluency was assessed by summing up all ideas. Elaboration was measured by rating the detailedness of each alternative ranging from 0 = *low detailedness*; 1 = *moderate detailedness*, and 2 = *high detailedness*. All answers were rated by two independent coders. Interrater-reliability (ICC estimates) and their 95% confidence intervals were calculated using SPSS (version 27.0, IBM, Armonk, NY, United States) based on a mean-rating (*k* = 2), consistency, and a two-way mixed-effects model. Results showed a moderate agreement between the raters, ICC (2, 172) = 0.68, *p* < 0.001, 95% CI [0.57, 0.77] (Koo and Li, 2016). The AUT is a reliable indicator to examine the creative potential of ideas (Dippo and Kudrowitz, 2013). Moreover, it can be understood as a performance measure as it is less affected by biases such as social desirability.

##### Perceived autonomy support

We used a self-translated, six-item version of the learning climate questionnaire (LCQ; Black and Deci, 2000). A sample item is: “I feel that my facilitator provides me choices and options” (α = *0.85*). Responses were collected using a 7-point scale ranging from 1 = *strongly disagree* to 7 = *strongly agree*.

##### Satisfaction with teamwork

Participants were asked “How satisfied are you with the outcome of your teamwork.” Responses could range from 1 = *very unsatisfied* to 10 = *very satisfied*.

##### Task and relationship conflict

We used the German version (Lehmann-Willenbrock et al., 2011) of Jehn’s (1995) intragroup conflict scale. Sample items include “How much tension is there among members in your team?” (*relationship conflict*; 4 items; α = 0.88) and “How frequently are there conflicts about ideas in your work unit?” (*task conflict*; 4 items; α = 0.88). Answers were given on a 6-point Likert scale ranging from 1 = *never/none* to 6 = *very often/very much*.

#### Data analysis

Due to the nested data structure, we applied multi-level analysis in R 4.02 using the package nlme (R Core Team, 2021; Pinheiro et al., 2022). The correlation between our two predictors, dominant regulatory focus (RFI) and dominant motivational index (MI) shows a negative non-significant correlation (*r* = −0.11, *p* = 0.15). We discuss on that in the discussion section. Team was used as a cluster variable. We centered RFI and MI and created a multiplicative interaction term using these centered variables to improve interpretation of parameters and to reduce risk of multicollinearity (Bickel, 2007). This is our model specification: Y = β_0_ + β_1_ RFI + β_2_ MI + β_3_ RFI × MI + e.^2^ To investigate significant interaction effects we used the package reghelper (Hughes and Beiner, 2021).

### Results

Means, standard deviations, and correlations between all variables are shown in Table 1. Estimates for RFI, MI and their interaction for all dependent variables are shown in Table 3.

**TABLE 3:** Estimates for RFI, MI and their interaction (with robust standard errors) for Study 1.

Variable	Predictor	Coefficient	*SE*	*t*	*p*
Fluency					
	**RFI**	**0.46**	**0.14**	**3.15**	**0.002**
	MI	−0.37	3.90	−0.09	0.925
	RFI × MI	−0.26	1.66	−0.16	0.874
Elaboration					
	**RFI**	−**0.03**	**0.01**	−**2.21**	**0.029**
	**MI**	−**0.97**	**0.33**	−**2.93**	**0.004**
	**RFI** × **MI**	**0.30**	**0.14**	**2.18**	**0.031**
Perceived autonomy support					
	RFI	0.05	0.04	1.41	0.162
	MI	−1.51	0.98	−1.55	0.123
	**RFI** × **MI**	**0.80**	**0.42**	**1.92**	**0.058**
Satisfaction					
	RFI	0.08	0.09	0.84	0.403
	MI	−4.09	2.58	−1.58	0.116
	**RFI** × **MI**	**2.19**	**1.10**	**2.00**	**0.048**
Relationship conflict					
	**RFI**	**0.09**	**0.04**	**1.93**	**0.056**
	MI	1.27	1.25	1.02	0.310
	**RFI** × **MI**	−**0.94**	**0.51**	−**1.82**	**0.072**
Task conflict					
	RFI	0.04	0.04	0.83	0.405
	MI	1.28	1.18	1.09	0.279
	RFI × MI	−0.63	0.49	−1.29	0.200

Significant effects at *p* < 0.05 and effects that reached a significance level of *p* < 0.10 are in bold.

We found support for hypothesis 1a and 2a, expecting main effects of RFI. More concretely, individuals high on promotion showed more fluency than individuals high on prevention and individuals high on prevention showed more elaboration than individuals high on promotion. We also found support for hypothesis 2b as there was a significant main effect of MI. Individuals high on avoidance motivation showed more elaboration than individuals high on approach motivation. We did not find support for hypothesis 1b, as individuals high on approach compared to avoidance motivation did not show more fluency. We did not find support for hypothesis 3, expecting a fit effect on fluency for people high on promotion and approach motivation.

**FIGURE 2:**
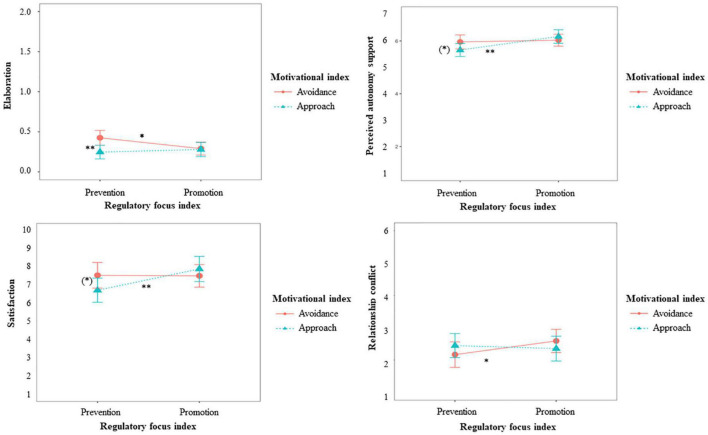
Effects of RFI and MI on elaboration, perceived autonomy support, satisfaction, and relationship conflict for Study 1. (*)*p* < 0.10, *p > 0.05, **p > 0.01. Elaboration: Simple slopes showed that for individuals high on promotion, approach or avoidance did not make a significant difference (*p* = *0.824*), but individuals high on prevention (*p* = 0.004) elaborated the ideas in more detail when they were high on avoidance compared to high on approach. For individuals high on approach (*p* = 0.539) promotion compared to prevention focus did not make a significant difference. Individuals high on avoidance (*p* = 0.012) elaborated the ideas in more detail when they were high on prevention compared to high on promotion. Perceived autonomy support: Simple slopes showed that for individuals high on promotion (*p* = 0.386), approach or avoidance did not make a significant difference. Individuals high on prevention (*p* = 0.088) tended to feel more supported in their autonomy when they were high on avoidance compared to high on approach. Individuals high on approach (*p* = 0.004) felt more supported in their autonomy when they were high on promotion compared to high on prevention. For individuals high on avoidance (*p* = 0.631), promotion or prevention did not make a significant difference. Satisfaction: Simple slopes showed that for individuals high on promotion (*p* = 0.371), approach or avoidance did not make a significant difference, but individuals high on prevention (*p* = 0.081) tended to be more satisfied when they were high on avoidance compared to high on approach. Individuals high on approach (*p* = 0.010) were more satisfied with the ideas when they were high on promotion compared to high on prevention. For individuals high on avoidance (*p* = 0.990), promotion or prevention did not make a significant difference. Relationship conflict: Simple slopes showed that neither for individuals high on promotion (*p* = 0.253), nor for individuals high on prevention (*p* = 0.220), approach or avoidance made a difference. For individuals high on approach (*p* = 0.627), promotion or prevention did not make a difference, but individuals high on avoidance (*p* = 0.030) perceived less relationship conflicts when they were also high on prevention compared to high on promotion.

We found support for hypothesis 4 (Simple Slopes are depicted and described in Figure 2), expecting a fit effect on elaboration for people high on prevention and avoidance motivation. More concretely, there was a significant interaction between RFI and MI, such that individuals high on prevention showed more elaboration when they were also high on avoidance motivation than when they were high on approach motivation.

For hypothesis 5 and 6, expecting fit effects on individuals’ general evaluation of the situation, we found partial support. For *satisfaction with the teamwork*, we found the expected interaction between RFI and MI (Simple Slopes are depicted and described in Figure 2). Individuals high on prevention tended to be more satisfied when they were also high on avoidance, providing evidence for hypothesis 6b. Approach motivation, on the other hand, was more beneficial for individuals high on promotion than for individuals high on prevention, providing evidence for hypothesis 5b.

For *perceived autonomy support* and *perceived relationship conflicts*, the interaction between RFI and MI failed to reach the significance level of 5%. However, the simple slopes are in the expected direction, not fully rejecting hypotheses 5a, 6a, and 5c (Simple Slopes are depicted and described in Figure 2). For perceived autonomy support, individuals high on prevention tended to feel more supported in their autonomy when they were also high an avoidance. Approach motivation, on the other hand, was more beneficial for individuals high on promotion than for individuals high on prevention. For relationship conflicts, avoidance seems to be more beneficial for individuals high on prevention. When those are avoidance motivated, they experience less conflicts than when individuals high on promotion are avoidance motivated.

Summarized, the study shows fit effects between regulatory focus and motivation for people’s elaboration of the ideas and the evaluation of the situation they were in. That is, preventers elaborated the ideas in more detail, were more satisfied and felt more supported in their autonomy when they were avoidance motivated than when they were approach motivated. For promoters, on the other hand, being approach or avoidance motivated did not make a big difference.

In Study 1, we measured motivational direction using the line bisection task. First, we do not know whether this measure reflects dispositional or situational motivation, as studies find links with dispositional as well as situational approach-related measures (e.g., Drake and Myers, 2006; Nash et al., 2010; Wilkinson et al., 2010; Naylor et al., 2015). Second, in service of the applicability of the research, a manipulation would make it much more likely to be able to come up with concrete, applied suggestions for how to improve a creative process. Therefore, we ran Study 2 in which we manipulated motivational direction.

## Study 2

### Materials and methods

#### Procedure and sample

The study was part of the Social Psychology course for bachelor students in their second semester at the University of Salzburg. As part of course, they also had small practice groups, consisting of 11–24 participants, where they had one workshop day getting to know various creativity techniques. At the beginning of the workshop, the teacher introduced them into different creativity techniques. After the students built small teams of two to six individuals, they received the first part of the study answering questionnaires. Then, they practiced one creativity technique using a real-life challenge (e.g., “How can we create social fairness in online teaching?”). The challenges were previously collected with the help of the teacher in order to create a realistic learning environment. After working on the challenge, students received the second part of the study consisting of the manipulation and the questionnaire with the dependent variables.

Data was collected from 147 participants. As part 1 or part 2 of the questionnaire, and/or team membership was missing for some participants, our final sample consisted of *N* = 112 that worked in 28 teams that were nested in 7 workshops. Participants were predominantly female (*N* = 87) and the age ranged from 18 to 35 years (*M* = 21.03; *SD* = 2.72).

#### Manipulation and measures

At the beginning of the small-group work we measured participants’ dominant regulatory focus, motivational direction, and their handedness. After they were working on a real-life challenge, we conducted the same test as in Study 1, i.e., the alternative use test (AUT), to examine subjects’ idea elaboration and fluency. Unlike in Study 1, in Study 2 there was a manipulation of approach vs. avoidance within the AUT. Afterward, we assessed sex and age and the same dependent variables as in Study 1, i.e., perceived learning climate (autonomy support), satisfaction with the outcomes of the teamwork regarding the real-life challenge, and experienced conflicts within the team.

##### Dominant regulatory focus index

Promotion and prevention focus were measured using the 20-item scale by Sassenberg et al. (2012). It consists of the promotion subscale with 12 items (α = 0.78; e.g., “My motto is ‘Nothing ventured, nothing gained”) and the prevention subscale with 8 items (α = 0.70; e.g., “If I do not reach my goal, I am becoming nervous”). Participants had to indicate on a 7-point scale ranging from 1 = *not at all* to 7 = *very much* in how far each statement applies to them. As in Study 1, we built a regulatory focus index (RFI), with positive values indicating a relatively higher dominant promotion than prevention focus.

##### Motivational index

As in Study 1, participants completed the line bisection task, involving 8 staggered horizontal lines of different lengths on the computer screen. Participants were instructed to mark the middle of each line with the cursor. Analysis steps were the same as in Study 1. Positive values indicated relatively more rightward errors and thus, approach motivation (*M* = −0.26, *SD* = 1.30, α = 0.70).

##### Manipulation of approach and avoidance

We used the same task from the alternative use test (AUT; Guilford et al., 1960) as in Study 1 but we included the manipulation of approach and avoidance within this task. The instructions were similar to the ones Roskes et al. (2012) used in their research. They read as follows:

Approach: “On the next page, please write down as many uses for a paper clip as you can think of. Make sure that you generate as many good ideas as possible! Ideas are a dime a dozen, but a large number of good ideas is a basic prerequisite for innovation. You have 3 min to do this. When you click on “Next,” the 3 min will start.”

Avoidance: “On the next page, please write down as many uses for a paper clip as you can think of. Make sure that you generate as few bad ideas as possible. Ideas are a dime a dozen, but no or a small number of bad ideas is a basic prerequisite for innovation. You have 3 min to do this. When you click on “Next,” the 3 min will start.”

Neutral: “On the next page, please write down as many uses for a paper clip as you can think of. You have 3 min to do this. When you click on “Next,” the 3 min will start.”

On the next page, there was a blank field and for the approach and avoidance condition a reminder to “please take special care to produce as many good ideas as possible” (approach condition), or to “please take special care to produce as few bad ideas as possible” (avoidance condition).

##### Fluency and elaboration

Fluency was assessed by summing up all ideas. Elaboration was measured by rating the detailedness of each alternative ranging from 0 = *low detailedness*; 1 = *moderate detailedness*, and 2 = *high detailedness*. The answers were rated by two independent coders. Since the first coder was no longer available for a group that took place at the very end, we called in a third coder for this group. Interrater-reliability (ICC estimates) between coder 1 and coder 2, and coder 2 and coder 3, and their 95% confidence intervals were calculated using SPSS (version 27.0, IBM, Armonk, NY, United States) based on a mean-rating (*k* = 2), consistency, and a two-way mixed-effects model. Results showed a good agreement between coder 1 and 2, ICC (2, 84) = 0.77, *p* < 0.001, 95% CI [0.65, 0.85], and also between coder 2 and coder 3 (2, 24) = 0.81, *p* < 0.001, 95% CI [0.57, 0.92] (Koo and Li, 2016).

##### Perceived autonomy support

We used the same six items as in Study 1 (α = *0.87*). Responses were again made using a 7-point scale ranging from 1 = *strongly disagree* to 7 = *strongly agree*.

##### Satisfaction with teamwork

Participants were asked “How satisfied are you with the outcome of your teamwork.” Responses could range from 1 = *very unsatisfied* to 10 = *very satisfied*.

##### Task and relationship conflict

We used the same scale as in Study 1 (*relationship conflict*; 4 items; α = 0.91) and “How frequently are there conflicts about ideas in your work unit?” (*task conflict*; 4 items; α = 0.90). Answers were given on a 6-point Likert scale ranging from 1 = *never/none* to 6 = *very often/very much*.

#### Data analysis

Due to the nested data structure, we applied multi-level analysis in R 4.02 using the package nlme (R Core Team, 2021; Pinheiro et al., 2022). Team was used as a cluster variable. The correlation between our two predictors, dominant regulatory focus (RFI) and dominant motivational index (MI) shows a positive significant correlation (*r* = 0.23, *p* = 0.01). We performed the same analyses as in Study 1 using centered dominant regulatory focus (RFI) and centered dominant motivational index (MI) and the multiplicative interaction term as predictors. This is our model specification: Y = β_0_ + β_1_ RFI + β_2_ MI + β_3_ RFI × MI + e. ^3^ We dummy coded the three manipulations approach, avoidance, and neutral condition and performed separate analyses with the dummy variables instead of MI. The respective condition (coded as 1) is tested against the other two conditions (coded as 0). This is our model specification that we used for each of the three dummy variables: Y = β_0_ + β_1_ RFI + β_2_ dummy + β_3_ RFI × dummy + e. To investigate significant interaction effects we used the package reghelper (Hughes and Beiner, 2021).

### Results

Means, standard deviations, and correlations between all variables are shown in Table 2.

#### Results of regulatory focus index and approach and avoidance manipulation

Please see Table 4 for estimates for RFI, the dummy variables of the conditions, and their interaction for all dependent variables.

**TABLE 4:** Estimates for RFI, the dummy variable and their interaction for Study 2.

Variable	Predictor	Coefficient	*SE*	*t*	*P*
Fluency					
	**RFI**	**0.76**	**0.38**	**1.99**	**0.050**
	Approach	−0.08	0.74	−0.11	0.914
	RFI × Approach	−0.002	0.76	−0.002	0.998
Elaboration					
	RFI	−0.003	0.04	−0.08	0.938
	Approach	−0.02	0.08	−0.26	0.796
	RFI × Approach	−0.10	0.08	−1.25	0.215
Perceived autonomy support					
	RFI	−0.10	0.09	−1.12	0.267
	**Approach**	**0.46**	**0.17**	**2.66**	**0.009**
	RFI × Approach	0.30	0.18	1.67	0.100
Satisfaction					
	**RFI**	−**0.42**	**0.18**	−**2.28**	**0.026**
	Approach	0.02	0.36	0.05	0.961
	**RFI** × **Approach**	**0.77**	**0.37**	**2.07**	**0.042**
Relationship conflict					
	RFI	0.04	0.05	0.81	0.420
	**Approach**	−**0.17**	**0.10**	−**1.72**	**0.089**
	RFI × Approach	0.05	0.10	0.47	0.638
Task conflict					
	RFI	0.10	0.08	1.19	0.238
	Approach	−0.26	0.16	−1.60	0.114
	RFI × Approach	−0.06	0.17	−0.36	0.722
Fluency					
	**RFI**	**0.86**	**0.39**	**2.20**	**0.031**
	Avoidance	−1.08	0.68	−1.58	0.118
	RFI × Avoidance	−0.37	0.70	−0.54	0.594
Elaboration					
	RFI	−0.05	0.04	−1.12	0.264
	Avoidance	0.09	0.07	1.15	0.254
	RFI × Avoidance	0.07	0.08	0.87	0.386
Perceived autonomy support					
	RFI	0.05	0.09	0.51	0.611
	**Avoidance**	−**0.39**	**0.17**	−**2.35**	**0.021**
	**RFI** × **Avoidance**	−**0.32**	**0.17**	−**1.87**	**0.065**
Satisfaction					
	RFI	0.04	0.19	0.21	0.834
	Avoidance	0.01	0.34	0.04	0.967
	**RFI** × **Avoidance**	−**0.88**	**0.35**	−**2.53**	**0.013**
Relationship conflict					
	RFI	0.03	0.06	0.62	0.535
	Avoidance	0.08	0.10	0.82	0.414
	RFI × Avoidance	0.07	0.10	0.73	0.467
Task conflict					
	RFI	−0.02	0.09	−0.24	0.813
	**Avoidance**	**0.31**	**0.16**	**1.95**	**0.055**
	**RFI** × **Avoidance**	**0.36**	**0.16**	**2.31**	**0.023**
Fluency					
	RFI	0.58	0.43	1.36	0.178
	**Neutral**	**1.16**	**0.69**	**1.67**	**0.099**
	RFI × Neutral	0.22	0.66	0.33	0.740
Elaboration					
	RFI	−0.03	0.05	−0.74	0.464
	Neutral	−0.08	0.08	−1.06	0.293
	RFI × Neutral	0.03	0.07	0.38	0.705
Perceived autonomy support					
	RFI	−0.07	0.11	−0.68	0.496
	Neutral	−0.02	0.18	−0.10	0.918
	RFI × Neutral	0.07	0.16	0.40	0.694
Satisfaction					
	RFI	−0.32	0.22	−1.48	0.143
	Neutral	−0.06	0.39	−0.16	0.876
	RFI × Neutral	0.21	0.33	0.64	0.522
Relationship conflict					
	**RFI**	**0.10**	**0.06**	**1.68**	**0.096**
	Neutral	0.16	0.12	1.29	0.200
	RFI × Neutral	−0.11	0.09	−1.14	0.256
Task conflict					
	**RFI**	**0.21**	**0.10**	**2.07**	**0.041**
	Neutral	−0.02	0.19	−0.08	0.935
	RFI × Neutral	−0.25	0.15	−1.65	0.104

Significant effects at *p* < 0.05 and effects that reached a significance level of *p* < 0.10 are in bold.

We found support for hypothesis 1a, expecting that people high on promotion show more fluency. More concretely, we found main effects of RFI, such that individuals high on promotion showed more fluency than individuals high on prevention. We did not find support for hypothesis 1b as people in the manipulated approach compared to the other conditions did not show more fluency. We did not find support for hypothesis 2a and 2b, expecting that people high on prevention compared to promotion or people in the manipulated avoidance compared to the other conditions show more elaboration.

We did not find support for hypothesis 3 and 4, expecting a fit effect on fluency for people high on promotion and approach motivation and a fit effect on elaboration for people high on prevention and avoidance motivation. That is, we did not find the expected interactions between RFI and the conditions.

**FIGURE 3:**
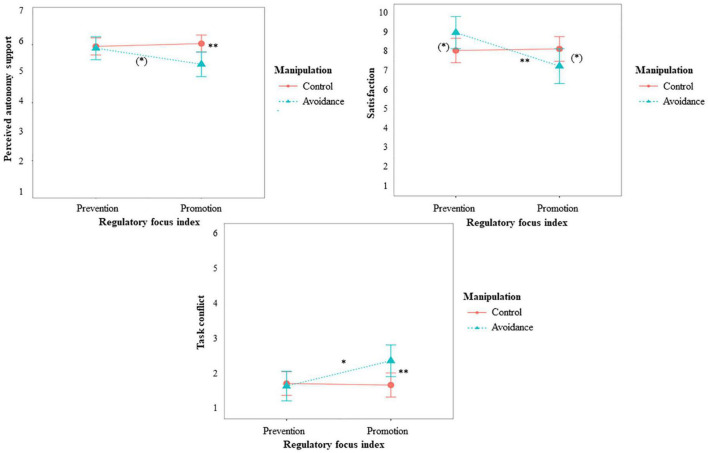
Effects of RFI and manipulated avoidance on perceived autonomy support, satisfaction, and task conflict for Study 2. (*)*p* < 0.10, *p > 0.05, **p > 0.01. Perceived autonomy support: Simple slopes showed that individuals high on promotion (*p* = 0.005) perceived lower autonomy support when they were high on avoidance, but for individuals high on prevention (*p* = 0.786) approach or avoidance did not make a difference. Individuals in the avoidance condition (*p* = 0.059) tended to be perceive more autonomy support when they were high on prevention than high on promotion. For individuals in the other conditions (*p* = 0.611), prevention or promotion did not make a significant difference. Satisfaction: Simple slopes showed that individuals high on promotion (*p* = 0.086) tended to be less satisfied when they were high on avoidance, and individuals high on prevention (*p* = 0.057) tended to be more satisfied when they were high on avoidance. Individuals in the avoidance condition (*p* = 0.005) were more satisfied when they were high on prevention than high on promotion. For individuals in the other conditions (*p* = 0.834), prevention or promotion did not make a significant difference. Task conflict: Simple slopes showed that individuals high on promotion (*p* = 0.005) perceived more conflicts when they were high on avoidance, but for individuals high on prevention (*p* = 0.748) approach or avoidance did not make a difference. Individuals in the avoidance condition (*p* = 0.010) perceived less conflicts when they were high on prevention than high on promotion. For individuals in the other conditions (*p* = 0.813), prevention or promotion did not make a significant difference.

**FIGURE 4:**
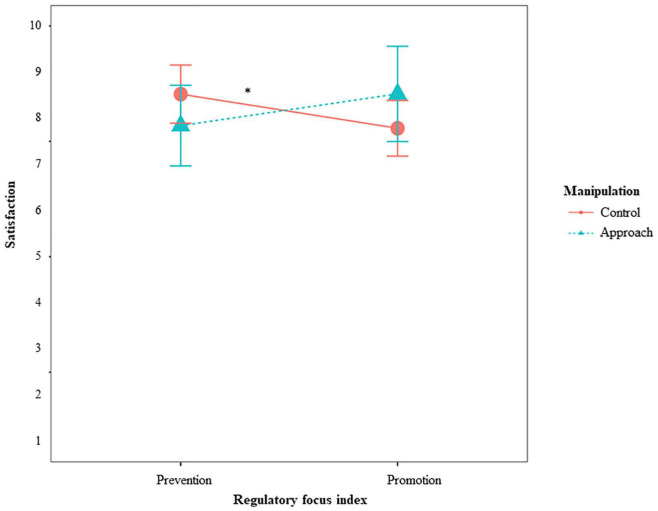
Effects of RFI and manipulated approach on satisfaction for Study 2. *p < 0.05. Simple slopes showed that neither for individuals high on promotion (*p* = 0.147), nor for individuals high on prevention (*p* = 0.122), approach or avoidance made a difference. For individuals in the approach condition (*p* = 0.286), promotion or prevention did not make a difference either but individuals in the other conditions were more satisfied when they were high on prevention than high on promotion (*p* = 0.026).

We found partial support for hypothesis 5 and 6 (Simple Slopes are depicted and described in Figure 3), expecting fit effects for individuals’ general evaluation of the situation. More concretely, for *satisfaction, perceived autonomy support*, and *task conflict*, we found the expected interaction between RFI and the avoidance condition. Individuals high on prevention were more satisfied when they were in the avoidance condition compared to the other conditions, providing evidence for hypothesis 6b. Individuals high on promotion were less satisfied when they were in the avoidance condition compared to the other conditions. Individuals high on promotion also perceived lower autonomy support and more conflicts when they were in the avoidance condition compared to the other conditions, providing evidence for hypothesis 5a and 5c. For individuals high on prevention, the condition did not make a difference. They experienced the same amount of autonomy support and task conflict, no matter which condition they were in. For the approach condition, promoters reported more satisfaction than preventers, providing evidence for hypothesis 5b (Simple Slopes are depicted and described in Figure 4).

Summarized, the study shows fit effects between regulatory focus and manipulated motivation for people’s evaluation of the situation they were in. That is, preventers were more satisfied when they were allowed to think about generating few bad ideas. Promoters on the other hand perceived the whole situation more negative, i.e., they were less satisfied, perceived less autonomy support and less conflicts, when they had to think about generating few bad ideas. They were more satisfied in the approach condition.

#### Results of regulatory focus index and motivational index

**FIGURE 5:**
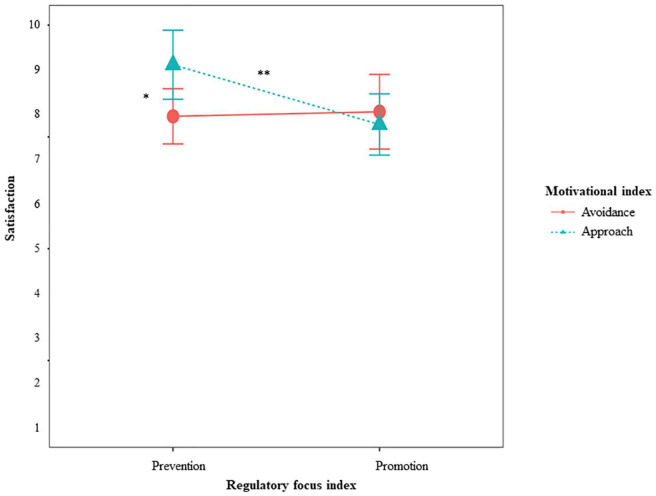
Effects of RFI and MI on satisfaction for Study 2. *p > 0.05, **p > 0.01. Simple slopes showed that for individuals high on promotion (*p* = 0.591), approach or avoidance did not make a significant difference, but individuals high on prevention (*p* = 0.010) were more satisfied when they were high on approach compared to high on avoidance. Individuals high on approach (*p* = 0.006) were more satisfied with the ideas when they were high on prevention compared to high on promotion. For individuals high on avoidance (*p* = 0.834), promotion or prevention did not make a significant difference.

We found some support for hypothesis 1a, expecting that individuals high on promotion show more fluency than individuals high on prevention, although the main effect of RFI on fluency failed to reach the significance level of 5%. For hypothesis 5 and 6, expecting fit effects on individuals’ general evaluation of the situation, we found an interaction between RFI and MI on satisfaction. However, the findings are in the opposite direction (Simple Slopes are depicted and described in Figure 5). Individuals high on prevention were more satisfied when they were also high on approach. Approach motivation, on the other hand, was more beneficial for individuals high on prevention than for individuals high on promotion, not providing evidence for hypothesis 5b nor for 6b.

We did not find support for the other hypotheses. Estimates for RFI, MI and their interaction for all dependent variables are shown in Table 5.

**TABLE 5:** Estimates for RFI, MI and their interaction for Study 2.

Variable	Predictor	Coefficient	*SE*	*t*	*P*
Fluency					
	**RFI**	**0.64**	**0.35**	**1.81**	**0.074**
	MI	−0.15	0.37	−0.41	0.680
	RFI × MI	−0.45	0.36	−1.25	0.214
Elaboration					
	RFI	−0.05	0.04	−0.133	0.187
	MI	0.04	0.04	1.03	0.307
	RFI × MI	−0.03	0.04	−0.73	0.469
Perceived autonomy support					
	RFI	−0.08	0.09	−0.85	0.395
	MI	0.11	0.09	1.23	0.221
	RFI × MI	−0.01	0.09	−0.13	0.899
Satisfaction					
	**RFI**	−**0.43**	**0.17**	−**2.49**	**0.015**
	MI	0.22	0.18	1.22	0.225
	**RFI** × **MI**	−**0.37**	**0.18**	−**2.10**	**0.039**
Relationship conflict					
	RFI	0.07	0.05	1.35	0.179
	MI	0.06	0.05	1.09	0.277
	RFI × MI	0.05	0.05	1.02	0.309
Task conflict					
	RFI	0.11	0.08	1.39	0.167
	MI	0.01	0.09	0.09	0.929
	RFI × MI	0.07	0.08	0.81	0.420

Significant effects at *p* < 0.05 and effects that reached a significance level of *p* < 0.10 are in bold.

## General discussion

This article investigated regulatory focus and fit by combining individuals’ dominant focus with their motivational direction to measure the outcomes of creative work. In Study 1, we measured regulatory focus as well as motivational direction and in Study 2, we measured regulatory focus and manipulated motivational direction. Both studies were performed within the context of creativity workshops and looked at individuals’ creative performance, i.e., fluency and elaboration, as well as their experience of the workshop. In both studies, we replicated the finding that a promotion focus is associated with fluency (Crowe and Higgins, 1997; Friedman and Förster, 2001; Liberman et al., 2001; Rietzschel, 2011). In Study 1 we also found that a prevention focus, as well as measured avoidance motivation were both associated with elaborated ideas. These main effects in Study 1 were qualified by an interaction of prevention × avoidance, indicating that preventers who were avoidance motivated elaborated ideas in more detail than preventers who were approach motivated. We did not find a promotion x approach fit for participants’ fluency. For individuals’ creative experience in Study 1, a fit between prevention focus and measured avoidance motivation resulted in a more positive experience of the workshop displayed by more satisfaction with the outcomes of the teamwork and more perceived autonomy support than a non-fit.

In Study 2, we did not find a fit effect on creative behavior. However, we found that the avoidance manipulation was unfavorable for the promoters’ creative experience: Promoters in the avoidance manipulation reported lower autonomy support, lower satisfaction, and more perceived conflicts within their teams than promoters in the approach condition or a neutral condition. The simple instruction to generate as few bad ideas as possible seems to make promoters feel uncomfortable. Interpreting it the other way round, this may mean that promoters feel better when they are given an approach-related instruction or a neutral instruction. One explanation for this finding may be that by the avoidance-based instruction, promoters are prevented from growing beyond themselves. An instruction to generate a large number of good ideas or even a neutral instruction may increase their hope for success which may better serve their desire for growth. As RFT (Higgins, 1997) claims, preventers on the other hand need to be safe. The instruction to generate a small number of bad ideas may decrease their fear of failure which may better serve their desire for safety. Testing hope for success vs. fear of failure as mediators within the creative process would provide us with more insights into the mechanisms.

For the results of measuring motivational direction in Study 2, we found that a non-fit, i.e., an interaction between prevention and measured approach, resulted in more satisfaction than a fit, i.e., an interaction between prevention and measured avoidance. Thus, preventers who were approach motivated reported more satisfaction with the teamwork than preventers who were avoidance motivated. This was surprising as we found the opposite pattern when measuring motivational direction in Study 1 and when manipulating motivational direction in Study 2. Various explanations are conceivable. One question is how valid and reliable the line bisection task actually is. What does it measure exactly? Is it approach or avoidance as a trait or as a state? The manipulation in Study 2 establishes approach and avoidance situationally, i.e., approach and avoidance are actually two strategies in Study 2. As expected and as predicted by the theory (Scholer and Higgins, 2008), fit effects are beneficial because the preferred strategies serve the respective system. If the line bisection task captures motivation situationally, we would expect that fit effects are beneficial. This has been shown in Study 1. Whether a fit between the systems or a non-fit between the systems is beneficial for individuals’ experience would depend on the situation people are in (cf. Lewin, 1943 field theory). In Study 2, people are involuntarily in the workshop because the workshop is part of a mandatory bachelor course for psychology students in their second semester. Promoters may see the workshop not as relevant to their growth goal, since it is a mandatory course and they do not see an opportunity here to act out their urge to grow and learn something new. For preventers who have their duties and tasks in mind anyway, just like attending the mandatory workshop, the workshop therefore has something positive for them and they see the workshop as something from which they can benefit. This becomes visible in the main effect of Study 2 that preventers are generally more satisfied with the outcomes of the teamwork than promoters. The interaction with measured motivational direction shows that they are even more satisfied when they are motivated by approaching their desired end-state of safety (0) than avoiding their undesired end-state of loss (−1). Thus, preventers who experience the workshop as a benefit, i.e., who are approach motivated, are more satisfied than preventers who experience the workshop as a loss, i.e., who are avoidance motivated.

Although one may assume that open-ended tasks that ask participants to connect new insights and create new solutions, such as creativity workshops, should rather fit individuals with a strong promotion focus (Faddegon et al., 2009), our results demonstrate that a prevention x avoidance fit could also benefit creative tasks. Interestingly, while for individuals high on prevention the fit could be shown in their performance and experience, for individuals high on promotion the fit was only visible in their experience of the situation. One explanation for the missing fit on promoters‘ behavior is that the creativity workshop is already a promotion-focused situation, so individuals high on promotion generate more ideas in general, independent of their motivation. This becomes visible by the main effect of promotion on fluency: In both studies, individuals high on promotion generated more ideas than individuals high on prevention.

Although the findings of the two studies are not consistent for all dependent variables, our studies bring forth an important message: For creative situations in which individuals work together on different tasks, a fit, i.e., promotion and approach or prevention and avoidance, seems to be more advantageous than a non-fit, i.e., promotion and avoidance or prevention and approach. This is especially relevant for situations in which one is able to actively induce a fit or non-fit (see section “Practical implications”).

### Theoretical implications

Overall, our studies contribute toward a better understanding of how motivational orientations impact creative work in several ways. First, the current studies replicate previous research showing that promoters are more fluent in generating new ideas (Crowe and Higgins, 1997; Liberman et al., 2001; Rietzschel, 2011). Second, the current studies extend regulatory fit research by considering the motivational direction and thus, creating a fit between people’s regulatory focus and their approach vs. avoidance motivation. Considering people’s motivational direction in creative work may not only lead to positive effects on emotional, motivational, and behavioral outcomes. It may also be a promising approach for understanding creative processes, i.e., their outcomes, their underlying mechanisms, and the interactive dynamics between people guiding the creative process, such as creativity trainers, and the people involved in the creative process.

Although a promotion focus is more strongly associated with creativity than a prevention focus, studies have shown that certain circumstances also boost creativity of prevention-focused individuals. For example, Baas et al. (2011) have shown that prevention-focused states that activate an individual can increase fluency and originality to almost the same level as promotion-focused states. When people in a prevention-focused state did not have closure, i.e., their goals were unattained, they were activated which resulted in original, creative ideas. However, a fulfilled goal deactivates people in a prevention focus as this leads them to feel relieved. In our Study 1, people with a dominant prevention focus were more creative (elaborated ideas) when they simultaneously were avoidance motivated. Avoidance motivation is associated with feelings of stress and anxiety (Elliot and Harackiewicz, 1996) which are also activated affects (Yik et al., 2011) and which are also core affects of a prevention focus when a goal is not fulfilled (Higgins, 1997). Thus, having an avoidance motivation may transfer people with a high prevention focus into a state of activation which positively affects their creativity. Nevertheless, this may have high cognitive costs for them (see Roskes et al., 2012). This reminds of work showing that if a task is impossible, a person’s motivational intensity is low. The person will not exert energy to take action on a goal (Brehm’s Motivational Intensity Theory; Brehm and Self, 1989; Wright, 2008). Transferred to our findings, this could mean that for preventers, the instruction to create many good ideas makes the task impossible for them. They need to have in mind not to create bad ideas which makes the task possible. They experience a state of stress and anxiety which activates them. Thus, the key variable for preventers to boost their creativity may be “unpleasant activation” (see Yik et al., 2011, for the circumplex structure of core affect). However, this assumption needs further testing.

### Practical implications

Our studies provide several important implications for the field of motivation and innovation. In times, where change is the new normal, organizations increasingly rely on creative and agile work to foster innovation and organization development. To make these autonomous teams effective, team members need an autonomous self-regulation which in turn provokes intrinsic motivation beneficial for pursuing goals (Sheldon and Elliot, 1999; Deci and Ryan, 2000). To foster this autonomous self-regulation, innovation coaches or leaders can address team members’ regulatory focus by using focus-specific communication to enhance the value of what people are doing (Higgins, 2005; Cesario and Higgins, 2008). With regard to promotion-focused people, coaches or leaders should take care that they do not use avoidance-related strategies in the first place but instead use approach-inducing strategies. Underlining gains might be such a strategy. With regard to prevention-focused people, coaches or leaders might want to use avoidance-related strategies such as underlining that avoiding mistakes is important.

A strategy which is congruent with one‘s personal preferences leads people to experience autonomous self-regulation and thus, intrinsic motivation (Sheldon and Elliot, 1999; Deci and Ryan, 2000). For example, in situations where creative work is needed, the person guiding the creative process (e.g., a leader, trainer, or instructor) should develop an increased sensitivity toward the regulatory focus of people. Then, they may be able to address the different foci, for example by using approach- or avoidance-related strategies. Consequently, people are enabled an autonomous self-regulation and success is possible.

Being sensitive also for one’s own regulatory focus can help oneself to better understand the reasons for own and also for others’ cognitions and behaviors. This can help for example, leaders, trainers or people guiding a group, choosing appropriate tasks or methods for their employees, trainees, or their group in order to develop creative ideas. This does not mean that one should always apply just methods fitting the regulatory focus as both regulatory foci are also accompanied by disadvantages. For example, promoters set themselves very ambitious goals but they often have difficulties implementing them (Böhm et al., 2017) and difficulties persevering in the process when the likelihood of failure is high (Lam and Chiu, 2002). As they try to use all opportunities to achieve their goals, they may not always use the best opportunity (Sassenberg and Vliek, 2019). Furthermore, their abstract and global view of the world can lead them to miss important details (Scholer and Higgins, 2012). The preventers’ strong focus on security makes it difficult for them adapting to changes in their environment. As changes can lead to failure, they perceive them as risks and thus, they rather stick to the *status quo* (Scholer and Higgins, 2012). Using the advantages of both foci may also be a possibility. For example, when an employee is a promoter, such as Alex in our example, s/he may have lots of visions but they are not properly thought through and thus, difficult to achieve. Here, one can stress the importance of setting small steps and employ methods that support Alex in getting into a prevention focus.

But how can we find out people’s regulatory focus? Although it can be a reliable and useful method to employ diagnostic instruments assessing regulatory focus (e.g., Keller and Bless, 2006), it does not seem appropriate for all situations and cases. The disadvantages of diagnostic instruments are that they are time-consuming, people may shy away from filling in a questionnaire, or they may respond with social desirability. Moreover, although each person holds a dominant regulatory focus (Strauman, 1996; Cesario and Higgins, 2008), the focus can also vary situationally (Higgins, 1998; Shah et al., 1998). As questionnaires may only assess an individual’s dominant but not situation-specific focus, one might categorize the person and underestimate the influence of the actual situation. Another, maybe more appropriate possibility is that one pays close attention to specific keywords the individual uses. These keywords can express the person’s regulatory focus, such as “success, change, growth, opportunities, desires, visions” for a promotion and “failure, security, duties, risks, mistakes, rules” for a prevention focus. Finding out the individual’s focus may also be achieved by various other methods, such as asking the person to write down the first goal-related associations that come to mind.

### Limitations and future research

In our studies we carried out creativity workshops which are perfectly suited for employing other approaches to collect data than only questionnaires. With regard to our performance measures, we did not rely on self-reports but counted the number of generated ideas and used external people who rated participants’ ideas on how elaborated they were. Relationship conflict, perceived autonomy support, and satisfaction with the outcomes of the teamwork were assessed using questionnaires but especially for Study 1, a 3-day workshop provides the benefit that team members develop commitment to their ideas and have enough interaction to let conflicts emerge. Nonetheless, there are certainly limitations of the study.

As we test different dependent variables regarding creative performance and experience, we performed a correction for multiple comparisons. Controlling for the family-wise error rate, we applied the Bonferroni correction with an alpha level of 0.05 and 6 tests for creative performance (three hypothesis for the two performance measures fluency and elaboration) and 4 test for creative experience (one hypothesis for the four experience measures autonomy support, satisfaction, relationship conflicts, and task conflicts). This results in a corrected alpha level of *p* = 0.008 for creative performance and *p* = 0.013 for creative experience. Interpreting the findings with the corrected alpha level, in Study 1 the significant main effects for RFI on fluency and MI on elaboration remain, thus supporting Hypothesis 1a that a promotion compared to prevention focus leads to an enhanced generation of ideas and Hypothesis 2a that an avoidance compared to approach motivation leads to an enhanced elaboration of ideas. All other Hypotheses did not find support with the corrected alpha level. Applying this correction, many of our significant results disappear. The main effects for fluency and elaboration remain only in Study 1 and the fit effect only remains for satisfaction and only in Study 2 where we manipulated avoidance and approach. This of course challenges our hypotheses and a more powered new study examining the findings that were significant (without and with alpha correction) would provide more clarity.

Looking at the correlations between regulatory focus and approach/avoidance motivation measured with the line bisection task, there was a negative, non-significant correlation in Study 1 (*r* = −0.11, *p* = 0.15) but a significant positive correlation in Study 2 (*r* = 0.23, *p* = 0.01), indicating that the more promotion-oriented a person is, the more approach motivated. It might be possible that, in the two studies, the line bisection task measured approach and avoidance at different levels. The non-significant correlation in Study 1 may indicate separability of the two factors, i.e., that regulatory focus and motivational direction are two different constructs, which are orthogonal at the system level (Scholer and Higgins, 2008). The significant positive correlation in Study 2 may indicate approach and avoidance at the strategy level. Here, promoters rather pursue approach- than avoidance-related strategies and preventers rather pursue avoidance- than approach-related strategies (Scholer and Higgins, 2008). This correlational difference may be due to the time regulatory focus and motivational direction have been measured.

In Study 1, we assessed the two variables at the beginning of the first workshop day and performed the AUT, our central dependent variable, at the end of the second workshop day. One may criticize that the measures are unrelated to the task participants are asked to do later on. However, as our regulatory focus scale assesses dominant regulatory focus, it should not have changed from day 1 to day 2. The line bisection task has shown to be a dispositional as well as state measure (Nash et al., 2010). Thus, it might be possible, that people’s motivation has changed from day 1 to day 2 and that our motivation measure is basically a measure of the strength of participants’ approach or avoidance motivation at the time of measuring. Rather than indicating something about the dynamics of regulatory focus and the states people experience while making creative decisions and interacting with their team, our results might say more about the interaction between two dispositional measures taken ahead of the workshop. That is, a certain subset of people with a combination of scores on motivational measures taken ahead of the workshop, performed better and were more satisfied with their experience.

In Study 2, we assessed dominant regulatory focus at the beginning of the team work which was in the middle of the workshop, and manipulated motivational direction within the AUT after participants had been working on a real-life challenge. Here, the manipulation is definitely situational. In Study 2, we also measured motivational direction using the line bisection task. This time, we measured it at the beginning of the team work, which could reflect a situational variable. Surprisingly, we found an opposite fit effect, such that preventers were more satisfied when they were approach motivated than when they were avoidance motivated. There were no main nor other interaction effects (Table 5). Our findings for Study 1 should be interpreted with caution as we do not know whether the line bisection task indeed reflects situational motivation in our studies. Some studies find links of the line bisection task with dispositional or situational approach-related measures, others do not (e.g., Drake and Myers, 2006; Nash et al., 2010; Leggett et al., 2016). To prove its validity, this would need further elaboration and additional measures.

Regarding our dependent variables for creative performance, the AUT may be a measure in which elaborating on ideas would undermine the fluency scores and thus unsurprisingly, in both studies the scores correlate negatively. However, it has been shown that both fluency and elaboration are uniquely associated with important creativity outcomes (e.g., Dumas et al., 2021). Hence, we consider both scores as valuable although the AUT might prompt fluency more than elaboration. It may be criticized that the AUT instructions are rather promotion-oriented and it would be better if it said “generate many and elaborated ideas.” This could be considered in future studies examining regulatory focus when performing the AUT.

Regarding the sample size of both studies, we are aware that they are small. As we performed the studies within creativity workshops that went over a certain time – Study 1 was part of a design thinking workshop and Study 2 was part of a workshop within the Social Psychology course for bachelor students in their second semester – we were only able to receive the data from a certain number of participants. However, findings should be interpreted with this information in mind.

Although promotion and prevention plays out in how people think about gains and losses, the situations in our studies were devoid of any stakes. In our creativity workshops, there was nothing to gain or to lose. However, promoters are in general more focused on approaching gains, which could be good ideas in our studies. And preventers focus in general more on avoiding losses, which could be bad ideas in our studies.

As regulatory focus plays a crucial role also in teamwork and groups in general, future studies should more strongly consider the group-context. Our study is one of the few investigating regulatory fit in the group-context. However, in our study we focused on individual outcomes (individuals’ experience of the situation and their individual creative performance) and not on outcome variables of the team. A study on table football found that a collective fit, i.e., a team’s overall fit between the team members’ dominant regulatory focus and the task demands of a team role, predicted team success (Memmert et al., 2015). However, investigating romantic relationships, a study by Bohns et al. (2013) found that couples who pursued similar goals reported more relationship satisfaction when they had different foci. Thus, for creative processes, looking at team performance or team experience when the team members have different foci (heterogeneous team) or the same focus (homogenous team) may be of special interest.

Since, especially in counseling, the relationship between counselor (in our study, the creativity trainer) and client is one of the central factors predicting counseling success (e.g., Grawe, 2004; Baron and Morin, 2009; Wampold, 2015; De Haan et al., 2016; Graßmann et al., 2019), future studies should also consider the interpersonal fit between counselor and client. In leadership research, an interpersonal fit between leaders and their followers indicate that people feel more valued and leadership is perceived as more effective (for an overview see Sassenberg and Hamstra, 2017). Coaching research indicates that promoters evaluate a promotion-coach and preventers evaluate a prevention-coach more positively. They find coaches with the same focus more likeable, believe to benefit more from them, and trust them more (Mühlberger et al., 2021). Actual investigations of the collaboration of dyads in creativity workshops such as DT with the same or different foci are necessary.

### Conclusion

Our results indicate that creative processes can benefit from the fit between individuals’ dominant regulatory focus and their motivational direction. Creative performance can be facilitated and people’s positive experience of the situation can be enhanced.

## Data availability statement

The datasets presented in this study can be found in online repositories. The names of the repository/repositories and accession number(s) can be found below: https://osf.io/uhbcq/?view_only=4166764c01da44159cab6bd9f6ae8e51.

## Ethics statement

The studies involving human participants were reviewed and approved by Ethikkommission der Fakultät 2 der TU Braunschweig and Ethikkommission - Paris Lodron Universität Salzburg. The patients/participants provided their written informed consent to participate in this study.

## Author contributions

PE, SK, and CM contributed to the conception and design of the study. PE and CM organized the database. JM, DH, and CM performed the statistical analysis. CM wrote the manuscript. PE, JM, SK, and EJ gave feedback to the manuscript. All authors contributed to the article and approved the submitted version.

## Footnotes

1 The idea evaluation of the experts is the rating of the ideas that participants gave on the challenges of the design thinking workshop. The results for this variable are reported in the Supplementary material. There were other measures not linked to explicit hypotheses that we did not include into our analyses. These variables are described in detail in the Supplementary material.

2 To control for hemispheric differences arising from handedness (e.g., Drake and Myers, 2006; Agroskin et al., 2016), we assessed participants’ handedness using the Edinburgh Handedness Inventory (Oldfield, 1971, 8 items; α = *0.77*). To rule out that handedness was a confound, we tested a model in which handedness interacted with MI and with RFI. We also compared the fit indices of the models including handedness and the models without handedness. As these interactions were non-significant and the fit indices of the models without handedness were better, we performed our analyses without handedness.

3 As there was an outlier in the line bisection task, we performed our analyses with a sample of *N* = 111. As in Study 1, participants’ handedness score was assessed using the Edinburgh Handedness Inventory (Oldfield, 1971, 8 items; α = 0.76) to control for hemispheric differences (e.g., Drake and Myers, 2006; Agroskin et al., 2016). To rule out that handedness was a confound, we tested a model in which handedness interacted with MI and with RFI. We also compared the fit indices of the models including handedness and the models without handedness. As these interactions were non-significant and the fit indices of the models without handedness were better, we performed our analyses without handedness.
