# Supplementary material for: Focus meets motivation: When regulatory focus aligns with approach/avoidance motivation in creative processes
Source: Front Psychol. 2022 Aug 30;13:807875. doi: 10.3389/fpsyg.2022.807875 (PMC9468904; doi:10.3389/fpsyg.2022.807875)
Supplement: Supplementary file 1 [file Data_Sheet_1.docx]

Supplementary Material

**Measures**

We measured participants’ creative-self-efficacy at the beginning of the first workshop day. At the end of the first day, we measured students’ idea generation and optimism. At the beginning of day two, we collected participants’ tolerance for ambiguity and their general causality orientation style (autonomy, competence, and impersonal 378 orientation). At the end of day three, we collected the idea evaluation of the experts.

*Creative self-efficacy*. We used a three item, 7-point scale (1 = *not at all*; 7 = *very much*) developed by Tierney and Farmer (2002; German version, Herbig, 2008) to measure creative self-efficacy. A sample item is “I have confidence in my ability to solve problems creatively”.

*Idea generation.* We used three items to measure idea generation (Janssen, 2000; German version, Hüttges, 2010). A sample item is: “I create new ideas for difficult issues”. Responses were collected using a 5-point scale ranging from 1 = *strongly disagree* to 5 = *strongly agree*.

*Optimism*. We used the revised life orientation test (r-LOT; Scheier, Carver, & Bridges, 1994; German version, Glaesmer, Hoyer, Klotsche, & Herzberg, 2008) to measure optimism. Sample items include “In uncertain times, I usually expect the best.” (optimism) and “If something can go wrong for me, it will” (pessimism). Answers are provided on a 5-point scale ranging from 1 = *I disagree a lot* to 5 = *I agree a lot*.

*Tolerance for Ambiguity.* We used the eight-item uncertainty tolerance scale developed by Dalbert (1999) to measure tolerance for ambiguity. A sample item is “I like unexpected surprises” and participants should indicate the appropriateness of each statement on a six-point Likert scale ranging from 1 = *totally incorrect* to 6 = *totally correct*.

*Basic Psychological Need Scale / General causality orientation.* We used the eight situational vignettes from a German version (Scherhorn, Haas, Hellenthal, & Seibold, 2014) of the General Causality Orientations Scale (Deci & Ryan, 1985) to measure participants’ need for autonomy, competence, and impersonal orientation. Each vignette describes a situation that is followed by three possible reactions. Each of these reactions represents autonomy, competence, or impersonal orientation, respectively. Participants had to indicate how likely it would be that they execute the described reactions on a 7-point scale (1 = *highly unlikely* to 7 = very likely).

*Solution Index*. Each developed idea for the challenges of the DT training was evaluated by two judges that were either experts for the given challenge or experienced design thinking facilitators. Judges indicated a) In how far they like the idea (1 = *not at all*; 10 = *very much*; idea attractiveness) and b) In how far they think that the idea will be implemented (10 percent = *highly unlikely*; 100 percent = *very likely*; idea feasibility). In total, the ideas were judged by 10 raters. We built a Solution index multiplying attractiveness by feasibility.

**Results**

| **Table 1** |  |  |  |  |  |
| --- | --- | --- | --- | --- | --- |
| *Estimates for RFI, MI and their interaction for Solution Index in Study 1* | | | | | |
| Variable | Predictor | *Coefficient* | *SE* | t | p |
| **Solution Index** |  |  |  |  |  |
|  | RFI | 0.002 | 0.003 | 0.63 | .530 |
|  | MI | -0.04 | 0.09 | -0.45 | .653 |
|  | RFI x MI | 0.02 | 0.04 | 0.50 | .615 |

Notes. RFI = Regulatory Focus Index; MI = Motivational Index.
